# Agroforestry Adoption Decision in Green Growth Initiative Programs: Key Lessons from the Billion Trees Afforestation Project (BTAP)

**DOI:** 10.1007/s00267-023-01797-x

**Published:** 2023-02-09

**Authors:** Ayat Ullah, Ashok K. Mishra, Miroslava Bavorova

**Affiliations:** 1 Faculty of Tropical AgriSciences, Czech University of Life Sciences Prague, Kamycka 129, 16500 Praha-Suchdol, Czech Republic; 2 Morrison School of Agribusiness, W. P. Carey School of Business, Arizona State University, 7271 E Sonoran Arroyo Mall, Mesa, AZ 85212 USA

**Keywords:** Timely adoption, Agroforestry, Green growth initiative programs, Billion Trees Afforestation Project (BTAP), Plantation success

## Abstract

Adoption of agroforestry in the early spring under the green growth initiative programs, such as the Billion Trees Afforestation Project, has positively impacted crop productivity and plantation success in Pakistan. However, the timely adoption decision of agroforestry in the Hindu Kush Himalayan is still low, particularly among smallholders. Using a mixed-method approach, we examine the factors influencing smallholder households and community-level timely agroforestry adoption decisions. Findings show that the early and later decision-makers farmers had agricultural lands on riversides and primarily adopted agroforestry to protect their crops from devastating effects of winds and floods. In contrast, late adoption decision-makers adopted agroforestry for income diversification. Results of our logit model show that several household and community-level factors influence smallholders’ timely adoption of agroforestry. The factors that positively affect the timely adoption of agroforestry include age-related factors, education, and the establishment/existence of functional community-based organizations. In contrast, political conflicts and tenure insecurity negatively affect smallholders’ timely adoption of agroforestry. In-depth interviews with Village Development Committees members revealed that terrace farming, farms on riversides, communities without access to other energy sources, population growth, and low farm acreage ensured the timely adoption of agroforestry. The policy recommendations include strengthening collaborative efforts among community members, especially engaging educated old-aged farmers (elders of traditional communities) to increase adoption rates and land tenure security to ensure timely agroforestry adoption under the newly launched 10-BTAP.

## Introduction

Recent literature in agroforestry offers insights into practices’ advantages and the timely adoption of agroforestry practices (Kantar et al., 2016; Buermann et al., 2018; Romanova et al., 2022). The time of adoption of agroforestry (in the adoption paradigm namely early, later and late) is crucial in the success of tree plantation. The early adoption of agroforestry, those adopting planting of trees in early spring, can enhance the success of plantations projects (Sukhbaatar et al., 2020). The adoption of agroforestry programs and practices in the early stages of availability (or timely adoption of agroforestry) enhances income and improves the sustainability of smallholder farms. The diffusion of agroforestry by extension agents and adoption by farming households can make the production system more resilient (Tafere and Nigussie, 2018). In addition, agroforestry diffusion and adoption can help increase crop productivity and income of small farmers in flood-prone areas and stabilize the environment (Jha et al., 2021). The adoption of agroforestry depends on farming communities’ situations and decision-making processes, extension systems and policies, farming systems, and community-based organizations (Trozzo et al., 2021; Romanova et al., 2022). The existing extension system in Pakistan does not support the widespread timely adoption of agroforestry by farming communities and holds the impact necessary for solving food security and livelihood demands in rural areas (Baig et al., 2021). Recently the Government of Khyber Pakhtunkhwa (GoKP) initiated an agroforestry/farm forestry free plants distribution program. The program through public forestry extension agents under the Billion Trees Afforestation Project (BTAP) is still ongoing under the flag of the recently launched 10-BTAP Project of the Government of Pakistan (GoP).

The BTAP is achieving the objectives of climate-smart landscape restoration and management and improvements in rural people’s livelihoods (Zada et al., 2022). The provincial government implemented the BTAP in the entire province of Khyber Pakhtunkhwa. The diffusion of agroforestry among rural households and communities was an essential activity within the program (Ullah et al., 2022a). The main objectives regarding BTAP were that the agroforestry diffusion would only be successful if the stakeholders would ensure timely and effective community-level participation in decision-making events (Ullah et al., 2022a). It was believed that promoting the adoption of agroforestry through communities’ participation would achieve its intended benefits of landscape restoration and poverty reduction (Ullah et al., 2021a). In this connection, community participation was considered an inherent activity for the success of BTAP (Zada et al., 2021). Community involvement in agroforestry diffusion decision-making increases community acceptance among community members and promotes its adoption (Ota et al., 2020). Indeed, local communities are considered critical beneficiaries and facilitators of any landscape restoration project (Ullah et al., 2021a). In the study area and under the BTAP, the willow, Robinia, Poplar, and Ailanthus trees were the critical plants distributed under the flag of *farm forestry free distribution* among farming communities.

In the mountainous regions of Pakistan, the adoption of agroforestry is particularly low, especially in smallholder farming under the BTAP (Ullah et al., 2022a). Furthermore, there is a failure among agroforestry plantations primarily due to farmers’ adoption in late spring or summer (late adoption) and poor management of agroforestry plantation sites (Ullah et al., 2022b). Moreover, the late adoption of BTAP also affected the quality and growth of plants (Ullah et al., 2022a). To increase timely and sustainable agroforestry adoption, farmers need to understand the values of agroforestry adoption in the early spring season (Luoranen et al., 2018). The forest department needs to understand the requirements that can hinder or complement the timely diffusion of agroforestry (Ullah et al., 2022a). Additionally, farmers need to know how the program can support landscape restoration, crop production, and income. Extension programs have promoted the adoption of agroforestry within integrated maize-based farming systems in different parts of the world (Wambugu et al., 2011; Nyasimi et al., 2017; Yadav et al., 2021). Some studies (Reid, 2017; Do et al., 2020; Nath et al., 2020) have suggested farming communities’ involvement in decision-making in promoting agroforestry practices. Other studies (Valdivia et al., 2012; Brown et al., 2018) have suggested discussing key policies that hinder or encourage the adoption and impact of agroforestry. Yet other studies (Ajayi and Place, 2012; Brockington et al., 2016) have suggested the diffusion of agroforestry practices in an area that can play a specific role in facilitating economic development programs.

The discussion on ensuring the timely adoption of agroforestry is largely missing in the literature. Most studies in the literature on the factors affecting the adoption of agroforestry focus on socioeconomic factors (Amare and Darr, 2020; Koussihouèdé et al., 2020; Gosling et al., 2021), policy (Amadu et al., 2020), social capital (Amare and Darr, 2020; Ahmad et al., 2021), external agents (Brockington et al., 2016), and access to information by farming households (Arimi and Omoare, 2021). There are also some studies on the temporal aspects of agroforestry adoption, which discuss the effects of initial adoption on farmer’s retention of plants (Brockington et al., 2016), promotion of positive agro-ecological system (Rosati et al., 2021) and economic and social benefits to farming communities (Romanova et al., 2022). The success of the agroforestry program necessitates all the stakeholders, including farmers, project staff, policymakers, extension agents, and scientists. It requires identifying the critical barriers to farmers’ adoption of agroforestry in its initial stage in green growth initiative projects such as the BTAP. Farming communities in mountainous regions depend on agriculture for livelihood (Li et al., 2020) and are often confronted with poverty, small acreages, and scarce off-farm employment opportunities.

Therefore, the central hypothesis of this study is that the success of the diffusion of agroforestry programs requires farmers’ timely adoption. The timely adoption decisions can bring better outcomes for farming communities in the above region, the project staff, and the environment (Ofori et al., 2020). Therefore, there is a need to identify the factors affecting the timely adoption of agroforestry by incorporating qualitative and quantitative data. Furthermore, there is a lack of studies focusing on rough-terrain regions, like the mountainous regions of Pakistan, which tend to be isolated from cities and towns. To the best of our knowledge, no study has examined the factors affecting the timely adoption of agroforestry. Timely adoption in our research is in the context of the time of adoption in the adoption paradigm (namely early, later, and late adoption). Therefore, this study analyzes farmers’ socioeconomic, local and institutional characteristics affecting the timely adoption of agroforestry under the BTAP in Pakistan’s Hindu Kush Himalayan (HKH) mountainous communities.

The novel aspect of this study is that it presents a holistic approach to the factors affecting farmers’ decision to adopt agroforestry during a particular time in the BTAP. We group smallholders into three groups based on the time of adoption in the technology adoption cycle. These groups include *early adoption decision makers* – the first group of adopters who collected agroforestry plants on time between October and November of 2020. *Later adoption decision makers*– the second group of adopters collected plants in December 2020 and January 2021, and *late adoption decision makers*–the third group of adopters collected agroforestry plants considerably late in February and March of 2021. Data has focused on community and household levels, identifying the key factors that affect households’ and communities’ decisions to adopt agroforestry at a particular time under the BTAP. The findings from this study are expected to inform policymakers to improve the planning and implementation for farmers’ timely adoption of agroforestry for sustainable landscape restoration initiatives.

## Theoretical Framework

The theory of diffusion of innovations and adoption of new practices by farm households is discussed by Rogers (Rogers, 2003). The Rogers theory is the baseline for analyzing the extension-based new practices for farming households (Li et al., 2018). In Rogers’s theory, diffusion involves the transfer of new practices to farmers in the S-shaped function of time (Rogers, 2003). Most of the time, efforts to increase the diffusion of new practices fail due to many farm households’ late adoption decisions (Cafer and Rikoon, 2018). Failure to promote timely adoption decisions among farming households is primarily because of the lack of interaction between the extension agents and farmers (Voss et al., 2021). The weak interaction between the extension system and farmers results in farming households’ low motivation and poor decision-making, resulting in the late adoption and failure of new practices in farmers’ fields (Cafer and Rikoon, 2018; Voss et al., 2021). Information dissemination and negotiation are often required for the timely adoption decisions of new practices (Aker et al., 2016). In this connection, technical support and knowledge transfer through frequent extension contact and dissemination of adequate information by BTAP experts are critical to promoting farmers’ timely adoption decisions.

Furthermore, concerns about ethnic and political differences among community members and their role in delaying the diffusion of new practices remain largely unaddressed in the literature. Moreover, the need to address community differences in farmers’ adoption and diffusion of new practices remains largely unaddressed in the literature. This implies that predicting the barriers to timely adoption decisions of agroforestry under the newly introduced 10-BTAP remains vital. Such predictions can be made by evaluating BTAP for promoting decision-making about the timely adoption of agroforestry in 10-BTAP. Previous research shows socioeconomic and institutional factors influence the adoption and diffusion of new agricultural practices (Wainaina et al., 2016; Ochieng et al., 2021). Therefore, in the current study, we include several socioeconomic, farm, and related institutional factors that we hypothesized to be influencing timely decisions of farmers to adopt agroforestry in the HKH and diffusion process of the BTAP. There has been little effort to predict the timely adoption of agroforestry in the BTAP activity of farm forestry free distribution.

We, therefore, use Rogers’s diffusion of innovations theory and the S-shape diffusion curve to assess the factors that affected the time of the decision to adopt agroforestry in the BTAP. Rogers’s theory discusses different groups of adopters (pioneers, followers, and laggards), which are based not only on the time of adoption but also on the percentages of the people (in our case, farmers) in the population who adopt an innovation. Our groups (early adoption decision makers, later adoption decision makers, and late adoption decision makers) are based only on time. The time of the decision to adopt agroforestry within 10-BTAP depends on political and ethical support, extension contact, community policies, and knowledge of the adoption process. We collected data from household heads and VDCs members to test the hypothesis using the survey method.

## Methodology

### Study Area

**Fig. 1:**
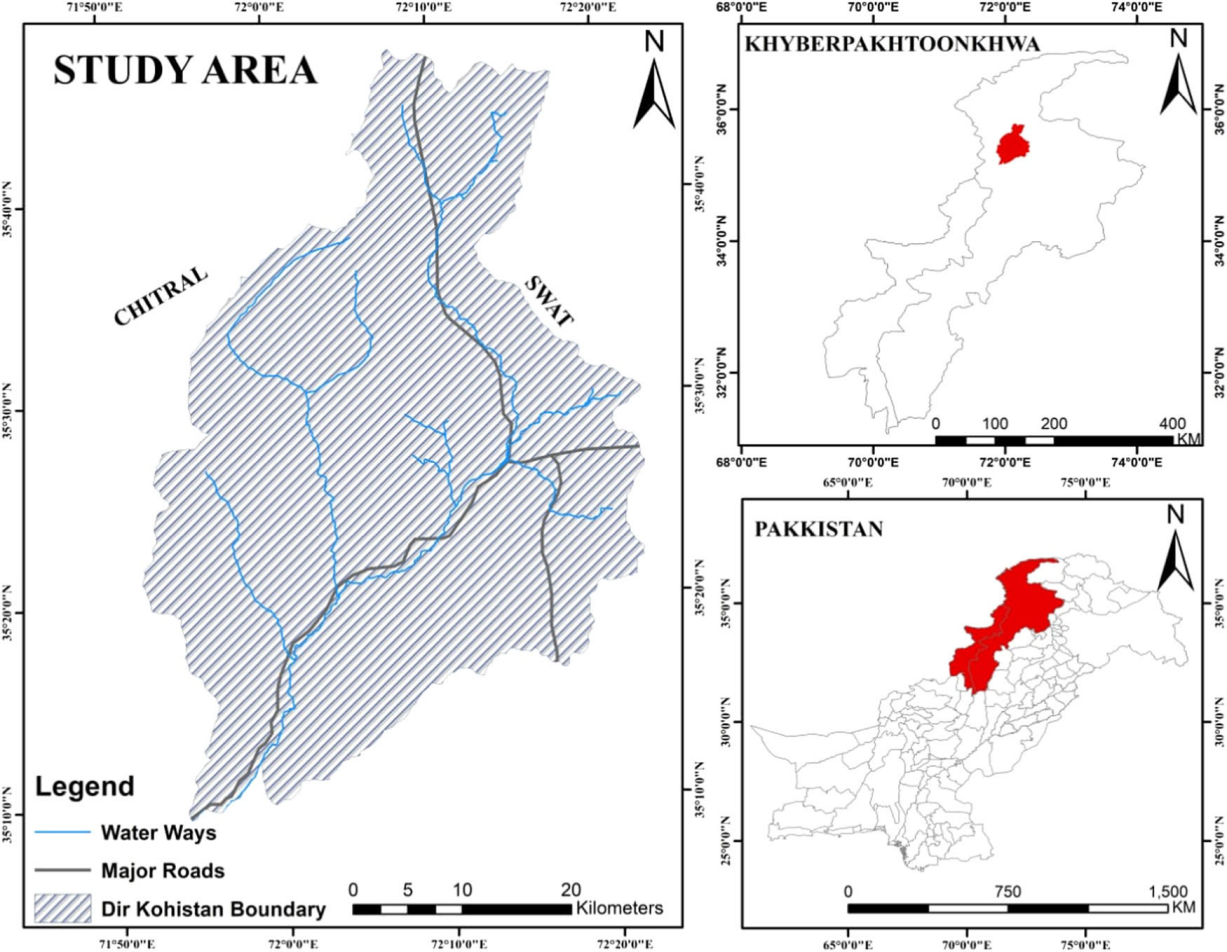
Study area

The Dir-Kohistan region has predominantly agricultural and heavily forested landscapes within Pakistan’s Hindu Kush Himalayan (HKH) region (Fig. 1). The HKH region is dominated by coniferous forests (Biland et al., 2021). The coniferous forest covers an area of 56,798 ha out of a total area of 167,032.39 ha (Ullah et al., 2022b). The local forest department manages these forests through community participation (VDCs) (Biland et al., 2021). Since the area is rich in forest lands, the Dir-Kohistan forest division was particularly important in the BTAP for restoration. Due to the small acreage and poverty conditions, the illegal timber trade threatens the entire valley. Farmers frequently convert forest lands into croplands to meet household subsistence needs (Zeb et al., 2019). Agriculture in the valley is primarily rainfed-dependent smallholder crop farming, considered a major livelihood activity. Major crops grown in the valley include wheat, potato, and maize. The fields are located mainly on the bank of River Panjkora and are exposed to frequent floods and droughts. Due to the high pressure on forests and reduced productivity of crop farming, the diffusion of agroforestry is significant activity in the valley. In connection with this, agroforestry diffusion was prioritized in the BTAP. The valley’s average annual minimum and maximum temperatures extend between 1000 mm and 1600 mm. The valley’s climate is relatively cold compared to the other terranean parts of Pakistan.

### Data Collection

Data for this study were collected from the forest and agricultural-dependent households and the Village Development Committees (VDCs) members through a detailed survey on a pre-constructed questionnaire. A total of 300 farm household heads and 60 VDCs members were surveyed using face-to-face interviews from February to September 2021. The government record showed that 8277 households participated in the agroforestry part of the BTAP from 2014 to 2019. Among them, a total of 3865143 free plants were distributed. The forest department also distributed plants in 2020 and 2021. Still, the number of households that participated and the plants distributed among them was unknown (the data for that time period was not entered in the official database). A total of 108 VDCs were active during data collection in the study region. As per the government policy, each VDC had 12 members. Plants for agroforestry were distributed among farm households on the recommendation of their VDC. The farmers were not obliged to report tree plantation and survival. However, the BTAP staff and VDCs used to check the plantation’s success and failure on farms. Moreover, the farmers were not strictly obliged to care for trees in the future.

The questionnaires contained closed and open questions, and the sampled respondents at the household level were key decision-makers of their respective families. The respondent VDCs members were principal decision-makers of their villages at the community level. All respondents for the study (from both groups) were selected randomly. The survey covered various topics related to the household’s socioeconomic characteristics, community/village-specific characteristics, community and households’ decisions on the adoption of agroforestry, involvement in BTAP planning and implementation, and the characteristics of local institutions in promoting agroforestry in the study area. A group of 15 trained enumerators gathered data in the local languages (Pashto and Kohistani) under the close supervision of the lead author. All enumerators were from study regions and were identical to local cultures and norms, and were graduates of forestry and agriculture departments. Each interview lasted from 45 to 60 minutes.

### Data Analysis

The statistical Package for social sciences (SPSS) version 25 was used to analyze the collected data. Both descriptive statistics, such as numbers and percentages, and regression analysis, such as the multinomial logit model, were used in the study. Descriptive statistics were used to analyze households’ demographic and socioeconomic characteristics such as age, education, household size, farming experience, landholding size, and community characteristics such as political and ethnic conflicts, community meetings, the establishment of community-based organizations such as village development committees (VDCs), extension contact and tenure insecurity. The multinomial logit model was used because households were divided into three groups (early adoption decision makers, later adoption decision makers, and late adoption decision makers). We use multinomial logistic regression to determine the factors affecting farmers’ early adoption of agroforestry in the BTAP (in the early adoption decision-makers group). Equation (1) estimates the probability of a household head *i* to adopt agroforestry at a specific time *j* in the free distribution of BTAP plants. The probability with which a household head *i* has a set of specific socioeconomic, community, and institutional characteristics *X*_*k*_ adopting agroforestry in the early stages of free distribution *j* was modeled as:

1 $$P\left( {Yi = j} \right) = \frac{{\exp \left( {\beta _j^\prime\, X_k} \right)}}{{\mathop {\sum}\nolimits_{j = 1}^3 {\exp \left( {\beta _j^\prime\, X_k} \right)} }},{{{\mathrm{Where}}}}\,j = 1,2,3\,{{{\mathrm{and}}}}\,X_k = X_1,X_2,X_3 \ldots \ldots \ldots .X_{12}$$

where *Y*_*i*_ is a dependent variable that is modeled into three levels of adopters groups *j* = 1; *j* = 2 and *j* = 3.

*j* = 1; Early adoption decision-makers – the first group of adopters includes households who collected agroforestry plants between October and November of 2020.

*j* = 2; Later adoption decision makers - the second group of adopters includes households who collected agroforestry plants between December 2020 and January 2021.

*j* = 3; Late adoption decision-makers–the third group of adopters, includes households that collected plants for agroforestry between February and March of 2021 in the BTAP. The characteristics of households’ head *i* is included in the multinomial logit model. These include household socioeconomic and community and institutional factors discussed in Section 2.4.

### Variables Used in the Study

#### Dependent Variable

We selected our dependent variable through the lead author’s observations and the farming communities’ complaints to the lead author about the poor quality and quantity of distributed plant species by some late adopters. This was because the lead author was part of the regional extension system, and all free distribution was conducted under his supervision. According to the lead author’s observation and routinely written and oral complaints by the late adopters, the forest department distributes healthy plants to farmers interested in early adoption. The species of interest and the species suited to local climatic conditions are allocated to early and later adoption decision-makers. Since the forest department was distributing plants for free, the early and some later adoption decision-makers collected more plants than they needed. The late adopters ordered significantly low quantities and quality of plants. Many late adopters stated that they required Populus trees; however, the forest department handover them Willow plants. Thus, we believe that farmers’ participation in early or late adoption influences their effective adoption of agroforestry. Therefore, we divided the adopters (our dependent variable) into three groups: early adoption decision makers, later adoption decision makers, and late adoption decision makers (Läpple and Van Rensburg, 2011). Out of a total of 300 farmers, 11.7% collected plants in October and November of 2020 (early adoption decision makers), 54.7% collected plants in December 2020 and January 2021 (later adoption decision makers), and 33.6% collected plants in February and March of 2021 (late adoption decision makers).

#### Independent Variable

Twelve factors were hypothesized in this study to affect farmers in one of the three categories. Farmers’ socioeconomic factors, such as the age of the households head, may affect a farmer’s decision to adopt agroforestry in the early stage of free distribution and be in the early or later adoption decision-makers category (Beyene et al., 2019; Le et al., 2021). The key reason is that older folks are decision-makers in the developing world, and therefore, they have better confidence than younger farmers (Singh et al., 2016). We expected that the educational attainment of the head of household help farmers adopt agroforestry in the early stages and, thus, influence farmers to be in the early or later adoption decision-makers category. Education increases the farmer’s awareness of the potential benefits of agroforestry adoption, and therefore educated farmers quickly adopt agroforestry for expected benefits (Fleming et al., 2019; Dhakal and Rai, 2020). Household size may enhance family’s demands for firewood and timber (Hussain et al., 2019; Bharadwaj et al., 2021). Thus, we expect the household size to positively correlate with a farmer in an early or later adoption decision-makers category. Farming experience enhances farmers’ understanding of the benefits of agroforestry adoption (Dhakal et al., 2015).

Increased experience positively correlated to the farmer’s early adoption decision (Dhakal et al., 2015). Agricultural land is considered a basic household asset for a farmer, and increased landholding size positively influences farmers’ early adoption of agroforestry (Dhakal et al., 2015; Beyene et al., 2019). Therefore, we hypothesize that the farm size positively affects a farmer’s early adoption decision. Findings by Lazos‐Chavero et al., (2016) and Corbera et al., (2019) suggest that the politically motivated conflicts in a community are critical determinants of a farmer’s timely adoption of agroforestry. In other words, differences in political interests in a community may establish a conflicting situation and thus may hinder members’ timely adoption of agroforestry (Lazos‐Chavero et al., 2016; Corbera et al., 2019). Therefore it is expected that the probability of adoption of agroforestry in the initial stages of a project is lower in politically divided communities and groups.

Similarly, members of a community divided into different ethnic groups and conflict groups will have poor adoption decisions, and the participation of such community members in the afforestation program will be low (Ullah et al., 2021a). Ethnic heterogeneity makes the communities vulnerable to several types of conflicts, especially regarding natural resources influences households’ timely adoption of agroforestry (Danquah, 2015). Thus, the ethnic diversity inside a community would establish conflicting situations among community members and, as a result, will negatively affect farmers’ decision to adopt agroforestry in the early or later adoption decision-makers group. Routine meetings of household’s critical decision-makers in the community during the season of agroforestry plantation increase the chances of farmer’s timely adoption of agroforestry (Bettinger et al., 2021).

Thus, we included the monthly or routine meetings of households’ key decision-makers during the plantation seasons in this study. Monthly meetings positively correlate with the farmers’ decision to adopt agroforestry and would determine if the farmer is an early or later adoption decision-maker. The farmers’ participation in afforestation and involvement in fast-growing tree plantations are negatively affected by elite capture (Ullah et al., 2022b). This study anticipated a negative correlation between farmers’ timely adoption of agroforestry plantations and the elite capture inside a community. We expect that in those communities where the elite capture is typical, the farmers will be in the late adoption decision-makers category in contrast to the early and later adoption decision-makers. Establishing functional VDCs is crucial for boosting farmers’ timely adoption of agroforestry (Ullah et al., 2021a). Therefore, we expected that the farmers could quickly adopt agroforestry in areas with functional VDCs. The farmers will be in early or later adoption decision-makers groups in such villages.

Farmer’s frequent contact with extension service providers or other forest department staff was positively correlated to their timely adoption of agroforestry (Ullah et al., 2021a). Farmers in close contact with the staff member of the forestry department (especially with extension agents) are more likely to engage in the timely adoption of agroforestry. Farmers in mountainous regions of Pakistan have low access to key assets such as land resources, forcing them to grow crops on a tenancy basis (Ullah et al., 2021b). Most farmers lease lands for a specific time, and these farmers do not know when the owners will take the land from them and hand it over to other tenant farmers. This affects farmers’ participation in agroforestry and afforestation activities (Ullah et al., 2021b). Therefore, our study expects a negative correlation between tenure insecurity and the farmers’ early adoption of agroforestry.

## Results and Discussion

### Descriptive Statistics

Descriptive statistics for explanatory variables used in this multinomial logit model are provided in Table 1. The average age of key decision-making farmer (usually elders of households) in the study region was about 47, with 28.74 years of farming experience. The average farm household size was about 13 members with 4.03 years of formal schooling. The farmers who adopted agroforestry under 10-BTAP had 3.00 acres of farmland. Among the farmers who adopted agroforestry under the 10-BTAP program, 57% reported political conflicts, whereas 46% of the respondents reported ethnic disputes in their communities. Among the respondents who have adopted agroforestry under 10-BTAP, about 30% reported monthly meetings of key decision-makers inside their communities. Similarly, 58% reported elite capture as a barrier to their timely adoption decisions among the adopters’ farmers. About 52% of agroforestry adopters’ reported functional VDCs in their communities. Of all farmers’ who adopted agroforestry under 10-BTAP, 20% reported frequent contact with the forest department officials, whereas 24% reported tenure insecurity as a barrier in their timely adoption decisions.

**Table 1:** Variables Definition and Descriptive Statistics

Variable	Description of Variables and Measurement	Mean (Std dev.)
Age	Age of a household head (in years)	47.06 (11.82)
Education	Education of a household head (in years)	4.03 (5.39)
Household size	Total number of family members in a household	12.46 (6.18)
Farming Experience	Total farming experience of the respondent (years)	28.74 (12.70)
Landholding size	Total agricultural land owned by a farmer (in acres)	3.00 (2.98)
Political conflicts	=1 if a household’s head adoption has been affected by politically motivated disputes during the last distribution season, 0 otherwise	0.57 (0.49)
Ethnic conflicts	=1 if a household’s head adoption has been affected by ethnic conflicts, 0 otherwise	0.46 (0.49)
Monthly meetings	=1 if monthly meetings of village key decision-makers are held during the planning of key activities or plantation season, 0 otherwise	0.30 (0.46)
Elite capture	=1 if a household’s head adoption has been affected by elite dominance in decision-making, 0 otherwise	0.58 (0.49)
Functional VDC	=1 if a functional VDC exists in a respondent village, 0 otherwise	0.52 (0.50)
Forest department contact	=1 if a household head is in frequent contact with the forest department, 0 otherwise	0.20 (0.40)
Tenure insecurity	=1 if a household head adoption has been affected by the insecurity of land tenure, 0 otherwise	0.24 (0.43)

Early adoption decision-makers = 35 (11.7%)

Later adoption decision-makers = 164 (54.7%)

Late adoption decision-makers = 101 (33.6%)

### Agroforestry’s Timely Adoption Benefits

**Fig. 2:**
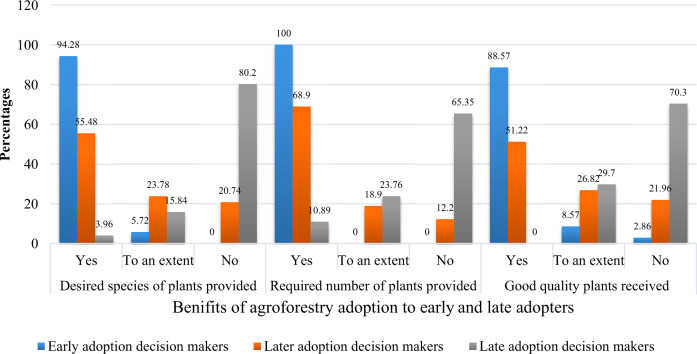
Comparison of adopters’ categories based on desired species, quality and the quantity of plants received

Figure 2 compares desired species collection in the required number and good quality among all three adopters’ categories. Figure 2 shows that the collection of selected species in the required number and sound quality is highest among early adoption decision makers, followed by the later adoption decision makers.

**Fig. 3:**
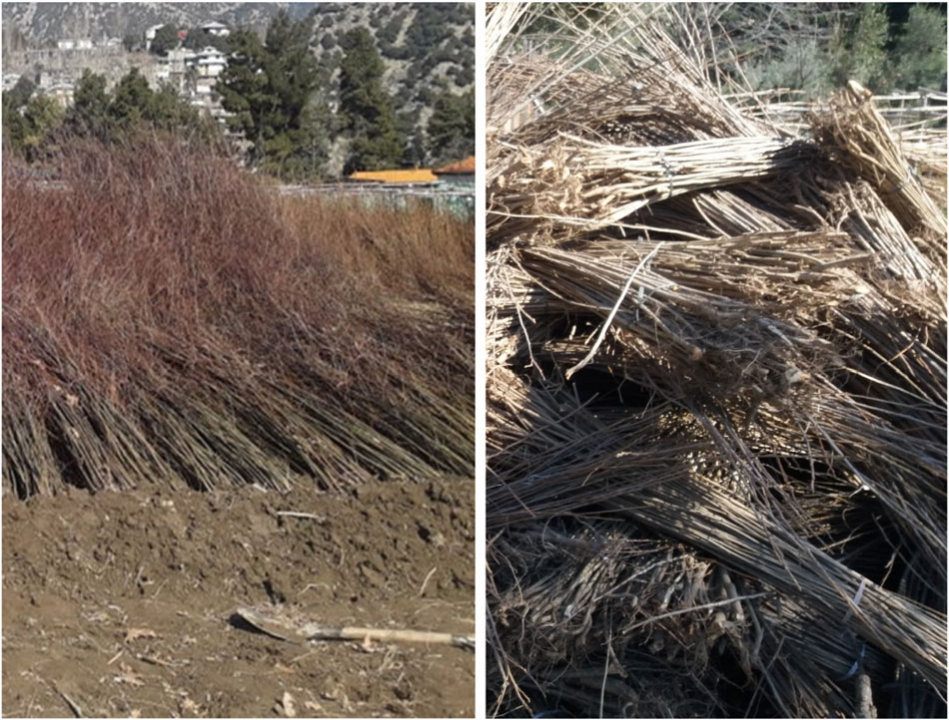
Quality of plants received by early (right) and late (left) adoption decision-makers

The early adoption decision makers have collected better plant species (Fig. 3) in maximum numbers than the late adopters. Most of the late adoption decision-makers, i.e., 70.3% (71 out of 101), reported that they had received poor quality plants (Fig. 2), and 65.35% of the respondents (66 out of 101) reported that they had not received plants in the required numbers (Fig. 2). Also, most late adoption decision-makers (80.2% or 81 out of 101) reported not being provided the desired species, which affected their complete adoption (Fig. 2). None of the late adoption decision-makers adopted the agroforestry program with full intensity under 10-BTAP. This suggests that the early adoption decision makers of agroforestry under BTAP were probably more effective and benefited more from the program than the late adoption decision makers.

### Factors Determining Farmers’ Early Adoption of Agroforestry

The study used multinomial logit (MNL) estimation procedure to assess the factors influencing the early adoption of agroforestry by smallholders. In other words, farmers are categorized into three groups. The early, later, and late decision-makers group. The late adoption decision-makers group is the base group. Variance inflation factors (VIFs) are the most common measure of assessing multicollinearity problems in independent variables (Esfandiari et al., 2020; Ullah et al., 2022c). The VIF values for all our tested explanatory variables were between 1.07 to 3.430, which specifies that all are below the threshold value of 10 (Kleinbaum et al., 2013). Thus, we found no multicollinearity problem in our variable. The value of pseudo-R^2^ was 0.446 implies that our model captures 44.60% of the possibility of farmers adopting agroforestry earlier (in early or later stages) and fits the data well. The LR Chi-Square statistic was significant at 1% (*P* < 0.05), indicating that some explanatory variables were maintained in the model, significantly affecting farmers’ adoption decisions.

The household head age is positively correlated with a farmer’s decision to adopt agroforestry at the beginning of free distribution and be in the early adoption decision-makers group compared to the group of late adoption decision-makers. Previous studies by Läpple et al., (2015) and Fisher et al., (2018) have provided contrary evidence to this finding. The authors found that farmers tended to use traditional farming methods with an increase in their ages. Thus, they were reluctant to adopt new agricultural practices. However, in our study, the early adoption decision-maker’s decision to adopt agroforestry in the early stages of free distribution partly explains two reasons. First, the old-aged farmers who choose to be early adoption decision makers over late adoption decision makers have complete control over lands and resources in the study region. Second, old-aged farmers are considered elders in the study region. They are the key decision-makers of all activities related to households and communities and were eager to restore their forests, agricultural land, and environments.

The education of a household head was significant and positively correlated with farmers’ decision to adopt agroforestry in the early stages of farm/agroforestry-free distribution. Thus, educated farmers are more likely to be early adoption decision-makers or later adoption decision-makers than late adoption decision-makers. This shows that a farmer with more education has adopted agroforestry earlier in the free distribution of farm trees under BTAP than the low-educated farmers. The apparent reason for educated farmers’ timely adoption of agroforestry could be that such farmers understand the benefits of early adoption. Moreover, knowledgeable farmers can make timely adoption decisions (Ofori et al., 2020). Our finding is consistent with Lienhoop and Brouwer (2015) and Singh et al., (2016), argue that educated farmers are more likely to be involved in timely adoption of agroforestry in afforestation projects.

Results show that political conflicts and divisions in farming communities have a negative and statistically significant effect on the early or later adoption decision-makers compared to the late adoption decision-makers. Results of our study show that politically motivated conflicts have negatively affected farmers’ decisions to adopt agroforestry. The possible reason could be that the politically motivated conflicts create division in the community. Such divisions and conflicts create unwillingness in farmers to collaborate with other farmers inside the community or with the agencies or departments promoting agroforestry. Many communities in the study area were divided into different political groups. They were not collaborating nor allowing the forest department to disseminate agroforestry effectively. Our finding is consistent with Barr and Sayer (2012) and Ullah et al., (2021a), who found that politically motivated conflicts create disputes over forest and land resources and affect the adoption of agroforestry.

Functional community-based organizations (CBOs) such as VDCs for developing farming communities at the village level is positively correlated with a farmer household’s decision to adopt agroforestry under BTAP well before the late and later adoption decision makers and be in the early adoption decision-makers group. This means that with a functional VDC in a village, farmers are more likely to adopt agroforestry in the BTAP in the early stage. This signifies the importance of functional community-based entities in playing an essential role in shaping community members’ adoption of new practices, such as agroforestry in the BTAP. Thus the establishment of functional VDCs in maximum villages will increase the chance of farmers’ timely adoption of agroforestry as it is the critical activity of BTAP. CBOs, such as VDCs at the household and community level, influence the adoption of agroforestry. The above organizations promote and encourage community members’ participation in the planning and implementing different project activities (Hendrickson and Corbera, 2015; Coulibaly et al., 2017). The positive correlation of CBOs, such as VDCs, with farmers’ timely adoption of agroforestry, is in line with the previous findings by Ullah et al., (2021a). Ullah et al., (2021a) reported higher and more timely adoption of agroforestry activities in villages with functional VDCs.

Our results suggest that the farmers’ decisions to adopt agroforestry in the later adoption decision-makers group are negatively affected by tenure insecurity. In other words, tenure-insecure farmers are less likely to adopt agroforestry in the later adoption decision-making group compared to the late adoption decision-making group of farmers. Thus, tenure insecurity has a significant and negative role in the farmers’ timely adoption of agroforestry. A possible reason could be that those farmers cultivating lands on a tenancy basis have customary insecure land rights in the study region. The area is mountainous, and farmers have low acreage. Many farmers rent land from other farmers to meet their households’ food and fodder needs, and they are usually afraid that the owner can take back his land anytime. On the other hand, agroforestry takes longer to provide income and firewood. This finding is consistent with Beyene et al., (2019), who indicated that tenure insecurity negatively affects Ethiopia’s timely adoption of agroforestry Table 2.

**Table 2:** Multinomial Logit Results for Farmers’ Early Adoption of Agroforestry

Variables	Categories of Agroforestry Adopters			
	Early Adoption Decision-Makers		Later Adoption Decision-Makers	
	B	S.E	B	S.E
Age	0.166^*^	0.053	−0.006	0.013
Education	0.532^*^	0.132	0.057^**^	0.032
Household size	0.035	0.058	−0.009	0.024
Farming Experience	0.010	0.028	0.006	0.011
Landholding size	0.106	0.124	−0.037	0.046
Political conflicts	−6.404^*^	1.810	−0.533	0.634
Ethnic conflicts	−1.188	0.923	−0.128	0.324
Monthly meetings	1.394	1.306	−0.061	0.307
Elite capture	−1.556	0.962	−0.112	0.299
Functional VDC	6.617^*^	1.804	−0.316	0.631
Forest department contact	0.497	1.554	−0.663	0.412
Tenure insecurity	0.073	1.247	−1.141^*^	0.426
	LLog-likelihood = 390.68, LR chi-square = 177.70, Chi-square sig = 0.000, Pseudo R2 = 0.447			

^*^Indicate significance level at 0.01, ^**^Indicate significance level at 0.10

Late adoption decision-making group is a reference category

### Primary Reasons for the Adoption of Agroforestry

**Fig. 4:**
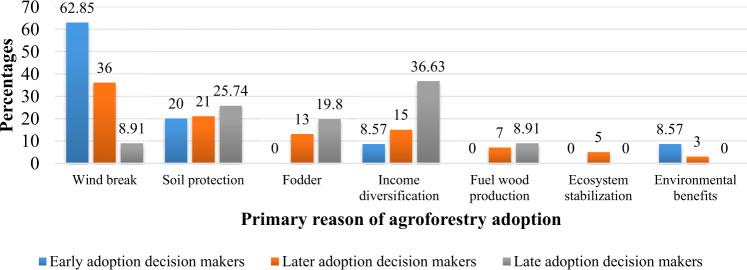
Comparison of adopters’ categories based on their specified primary reason for agroforestry adoption

For most early and later adoption decision-makers in the BTAP, the primary reason for adopting agroforestry was windbreak (Fig. 4). Many early and later adoption decision-makers reported *windbreaks as their primary reason for adopting agroforestry because agroforestry plays an essential role in crop protection from wind damage*. This means that compared to late-adoption decision-makers, the early and late adoption decision-makers have large acreages of croplands prone to wind damage. On the other hand, adopting agroforestry by late adoption decision-makers was an important economic activity primarily aimed at supporting profit from farm trees (Fig. 4). Many late adoption decision-makers reported that *to poor farmers adopting agroforestry is important for sustaining their livelihood and income*. Most farmers from the early adoption decision-makers group have reported windbreak followed by soil protection as their primary reason for adopting agroforestry. After windbreak and soil protection, the other important reason specified by farmers behind agroforestry adoption was income diversification, mentioned mainly by late adoption decision makers (Fig. 4).

### VDCs Members’ Perceptions of the Critical Factors Influencing Households’ Early Agroforestry Adoptions Decisions (from a Community Perceptive)

To know the complete pictures behind farmers’ early and/or late adoption, we interviewed VDCs members to learn about some additional reasons that we might not have covered in our households’ survey. For this reason, we first contacted 5 VDCs members. We listed key factors influencing the early or late adoption of agroforestry under the BTAP in the study region at the community level. After constructing a list of key variables, we interviewed 60 randomly selected VDCs members. Their joint statements are merged into one to reduce the respondents’ quotation, at the end of which the respondents are identified as tiny letters just above the line of text (Ullah et al., 2022d). Table 3 provides an overview of the hierarchy of different variables that shape the categorization of farmers in early, later, or late adoption decision-makers groups, which are discussed further below.

**Table 3:** VDCs Member Perceptions of Key Factors Influencing Communities’ Early Agroforestry Adoptions Decisions

Variables	VDCs Members	
	Cases	Percentage
Terrace farming	47	78.33
Farms at river banks	38	63.33
Communities have no access to other sources of energy	34	56.66
Population growth and low farm acreage	32	53.33
Livestock ownership	29	48.33

*N* = 60

#### Terrace Farming

Most of the VDCs members reported that the early adoption decision-makers usually reside in sloppy areas. Soil erosion is high from the agricultural fields in such villages, especially during rainfall, due to farming on terraces that are made with technical flaws. Also, they face frequent landslides due to slopes. The farmers in these villages consider agroforestry trees an important element that can control soil erosions and landslides and boost productivity on the farm. These villages consider agroforestry trees a critical factor in preventing soil erosions and landslides, thus increasing farm productivity. Early adoption decision makers do not adopt agroforestry for harvesting. Still, they consider it a necessary part of their agricultural system on the farm, and therefore, they are quick in adoption decisions. Many VDCs members reported that:

*Due to sloppy lands, soil erosions and landslides are common in our villages from agricultural fields. However, we cannot stop cultivation on derelict grounds in this region because we don’t have any other land available. Our village farmers believe that the timely adoption of agroforestry can prevent soil erosions and landslides. Therefore, many are early or later adoption decision makers in adopting agroforestry*.^1, 13, 24, 27, 45, 51^

Similarly, some VDCs members reported that:

*Agroforestry is not merely farming trees. As we grow crops of maize and wheat and consider them important for our lives, we grow trees on our farms and consider them important. We do not harvest trees for selling in markets. However, we consider them important for controlling soil erosion and increasing crop productivity. The agroforestry program under BTAP is how our community farmers think they can grow maximum trees on farms and, therefore, are early to make adoption decisions. We’re about improving our soil conditions and crop productivity, so we do not wait and adopt*. ^12, 22, 25, 31,42^

Various studies worldwide have reported that agroforestry control soil erosion and landslides which can lead to their early adoption in sloppy regions (Deb, 2020; Hairiah et al., 2020; Mishra et al., 2022).

#### Farms at River Banks

The other reason why some farmers were inclined to adopt agroforestry in the HKH region earlier than others was their cropland location. The VDCs members reported that the croplands of farmers from early adopters were on the river sides. Therefore, they adopted agroforestry earlier (on time) than late adopters. The VDCs members reported that the early adopters had experienced more flooding every rainy season, and they believe that agroforestry can help to slow the flow of water into the croplands. Many members of VDCs reported that:

*In BTAP, the early adoption decisions of agroforestry have gained more attention among farmers that have farmland on river banks*. ^2, 3, 17, 29, 55^

Similarly, some of the VDCs members reported:

*Early adopters of agroforestry were more convinced of the agroforestry adoption benefits than the late adopters. They had the prior understanding that the adoption of agroforestry could promote the sustainability of their croplands. They believed that agroforestry adoption could increase agricultural productivity by bringing efficiency in soil nutrients and water*. ^9, 14, 48^

This result confirms the findings of various previous studies (Dumont et al., 2019; Mahmood and Zubair, 2020; Hairiah et al., 2020), where the early adoption of agroforestry occurred among the farmers that had farmland beside rivers.

#### Communities Have No Access to other Sources of Energy

The other vital factors revealed by the VDCs members were the nonavailability of alternative energy sources, making communities overly dependent on the forest for meeting energy needs. In many communities, forests are critical for utilizing households’ energy needs, making the forest disappear quickly. In such communities, the farmers who depended solely on wood for energy started adopting agroforestry under BTAP. Moreover, such farmers were quicker to adopt agroforestry than the other farmers. Many VDCs members reported that:

*Adoption was too early in energy-deficient communities. Those communities dependent on forests for energy quickly adopted agroforestry under BTAP. They adopted it earlier than the other communities’ farmers with access to alternative energy sources. Early adoption of agroforestry promoted the sustainability of their forest lands. It reduces deforestation by reducing over-dependence on natural forests*. ^9, 31, 40, 56^

Similarly, some farmers reported that:

*Agroforestry adoption was earlier among wood-dependent communities. Heavy damage to natural forests and households’ intensive demand for firewood compiled communities to adopt agroforestry quickly and in large numbers so that they could use them as firewood in the future*. ^5, 7, 15, 30, 33, 41^

This result coincides with Nagar et al., (2022), highlighting that households’ dependence on firewood plays a crucial role in adopting agroforestry.

#### Population Growth and Low Farm Acreage

The results of interviews with VDCs members revealed that population growth and low farm acreage was other important component that promoted early and later adoption among farmers. They contended that low acreage was inefficient in fulfilling the growing populations’ livelihood needs and that farmers were collecting additional money from nearby forests. They realized that without adopting BTAP practices, they were further trapped in poverty. A VDC member reported:

*Because of low farm acreage and increasing population, farmers clear the forest for their subsistence. Many crops are grown for food, and the surplus is sold for cash; however, the income obtained from this was not enough for food and income in hilly areas. Seeing the increasing deforestation rate, the farmers stated agroforestry adoption under the BTAP*. ^56^

Another VDC member added that the crop grown in the mountainous areas were not sufficient, and with time, due to the increase in population, there was increased pressure on the forest for income. Despite the increased dependence of households on crops and forests, they were inefficient in alleviating household poverty. A VDC member who was a key decision-maker in his community reported:

*“Population growth and low farm acreage increased the pressure on forest income, resulting in rapid forest degradation. Agroforestry adoption helped to increase agricultural productivity and proved an alternative source of forest income. This was among the main reasons some farmers quickly adopted agroforestry under the BTAP.”* ^33^

Agroforestry adoption means a switch in land-use practices that may increase resilience toward floods and droughts and increase crop productivity and income (Simelton et al., 2015; Jha et al., 2021). Previous studies have reported that adopting agroforestry represents agricultural diversification and enhances crop production (Beillouin et al., 2021; Ndlovu and Borrass, 2021). Households that have perceived that forests have declined and that decline is due to deforestation and poverty, and they earn less crop-farm incomes mainly adopt agroforestry earlier resilience (Ruf and Schroth, 2015).

#### Livestock Ownership

In the mountainous regions of Pakistan, livestock is an integral part of households’ livelihood that has positively impacted the early adoption of agroforestry by community members. The VDCs members that promoted earlier adoption of agroforestry was household ownership of livestock. They used the newly adopted agroforestry systems for feeding livestock that reduced pressure on rangelands, fodder crops, and forest lands. A member of a VDC who was well-respected in his community reported:

*“Livestock is a key component in many communities. Early and some later decision-makers have the prior understanding that adopting agroforestry and forest trees such as willow, Robinia, Poplar and Ailanthus trees are used as a folder. They had the experience and knowledge that adopting agroforestry can help to reduce livestock-intensive grazing in forest areas. They were aware that agroforestry adoption can help them protect natural regenerations (due to reduced grazing) and forest conservations.”* ^21^

These results are similar to the previous findings of Jara-Rojas et al., (2020) and Beyene et al., (2019), who reported that livestock ownership influences the adoption of forest trees on farms.

## Conclusion and Policy Implication

The sustainable diffusion of agroforestry and its timely adoption by farm households plays a vital role in the success of green growth initiative projects. Farmers’ timely adoption of agroforestry can lead to a high success rate of plantations on farmers’ fields, climate risk mitigation, crop improvement, and income sustainability for smallholder farmers. However, little is known about whether agroforestry is disseminated in the desired manner by the forest department. Furthermore, there was little evidence of factors shaping farmers’ timely adoption of agroforestry under the BTAP.

Our estimates of descriptive statistics confirmed that all the early and most of the later adoption decision-makers had collected desired species of plants in good quality and required numbers. However, the late adopters have failed to collect the required species of plants in good quality and the numbers needed due to their late decision to adopt agroforestry. Findings from this study showed that farmers’ decisions to adopt agroforestry in the early group (timely adopters) are positively related to farmers’ age, education, and community-based organizations services such as VDCs in a”‘ farmer’s village. Similarly, farmers’ early and later adoption decisions were negatively correlated with political conflicts in the community and tenure insecurity. The negative and significant correlation indicated that political conflicts in the community and tenure insecurity have resulted in farmers’ late adoption decisions. In addition, our findings also revealed that most early and later adoption decision-makers had adopted agroforestry for reducing wind effects of slowing down wind speed. Similarly, most early adopters’ farms were on the river sides, and they adopted agroforestry early to prevent crops from flooding. In contrast, most late adoption decision-makers adopted agroforestry as an economic diversification strategy. Our in-depth interviews revealed that some community factors also affected farmers’ timely adoption of agroforestry besides household-level factors. These included communities on the terrace, farms on river banks, communities lacking other energy sources, population growth, small farm acreage, and high livestock owners. Thus, findings suggest that extension services should keep the above factors in mind while conducting diffusion of agroforestry

Our findings have important policy implications for the diffusion of sustainable agroforestry practices among smallholders in the HKH mountainous range. The timely adoption of agroforestry can improve the effectiveness of the agroforestry program. Therefore, it is essential to motivate farmers’ early adoption. Policies promoting the timely adoption of agroforestry should consider farmers’ decision-making processes and characteristics. For example, forestry extension agents can engage in informal educational campaigns in less educated farming communities and inform smallholders about the benefits of agroforestry in crop productivity and income. Similarly, villages divided into political grounds and disputes can be motivated for the timely adoption of agroforestry by establishing functional community-based organizations such as VDCs. The government could establish a policy for tenant farmers so tenure insecurity may not affect their timely adoption. Information dissemination on the timely adoption of agroforestry in the study region is needed. Finally, the household head’s age-related factors positively impact farmers’ timely adoption of agroforestry. Thus, the government could seek the help of elderly farmers in the diffusion of agroforestry.

## Data Availability

The authors confirm that the data supporting the findings of this study will be available from the corresponding author upon reasonable request.

## References

[CR1] Ahmad S, Caihong Z, Ekanayake EMBP (2021). Livelihood Improvement through Agroforestry Compared to Conventional Farming System: Evidence from Northern Irrigated Plain, Pakistan. Land.

[CR2] Ajayi OC, Place F (2012) Policy support for large-scale adoption of agroforestry practices: experience from Africa and Asia. In Agroforestry-The Future of Global Land Use (pp. 175–201). Springer, Dordrecht

[CR3] Aker JC, Ghosh I, Burrell J (2016). The promise (and pitfalls) of ICT for agriculture initiatives. Agric Econ.

[CR4] Amadu FO, Miller DC, McNamara PE (2020). Agroforestry as a pathway to agricultural yield impacts in climate-smart agriculture investments: Evidence from southern Malawi. Ecol Econ.

[CR5] Amare D, Darr D (2020). Agroforestry adoption as a systems concept: A review. For Policy Econ.

[CR6] Arimi K, Omoare A (2021). Motivating cocoa farmers to adopt agroforestry practices for mitigating climate change. Renew Agric Food Syst.

[CR7] Baig MB, Burgess PJ, Fike JH (2021). Agroforestry for healthy ecosystems: constraints, improvement strategies and extension in Pakistan. Agrofor Syst.

[CR8] Barr CM, Sayer JA (2012). The political economy of reforestation and forest restoration in Asia–Pacific: Critical issues for REDD+. Biol Conserv.

[CR9] Beillouin D, Ben-Ari T, Malézieux E, Seufert V, Makowski D (2021) Positive but variable effects of crop diversification on biodiversity and ecosystem services. Global Chang Biol 27(19):4697–4710 10.1111/gcb.15747 34114719

[CR10] Bettinger T, Cox D, Kuhar C, Leighty K (2021). Human engagement and great ape conservation in Africa. Am J Primatol.

[CR11] Beyene AD, Mekonnen A, Randall B, Deribe R (2019). Household level determinants of agroforestry practices adoption in rural Ethiopia. For, Trees Livelihoods.

[CR12] Bharadwaj B, Pullar D, To LS, Leary J (2021). Why firewood? Exploring the co-benefits, socio-ecological interactions and indigenous knowledge surrounding cooking practice in rural Nepal. Energy Res Soc Sci.

[CR13] Biland M, Zeb A, Ullah A, Kaechele H (2021). Why Do Households Depend on the Forest for Income? Analysis of Factors Influencing Households’ Decision-Making Behaviors. Sustainability.

[CR14] Brockington JD, Harris IM, Brook RM (2016). Beyond the project cycle: a medium-term evaluation of agroforestry adoption and diffusion in a south Indian village. Agrofor Syst.

[CR15] Brown SE, Miller DC, Ordonez PJ, Baylis K (2018). Evidence for the impacts of agroforestry on agricultural productivity, ecosystem services, and human well-being in high-income countries: a systematic map protocol. Environ Evid.

[CR16] Buermann W, Forkel M, O’sullivan M, Sitch S, Friedlingstein P, Haverd V, Richardson AD (2018). Widespread seasonal compensation effects of spring warming on northern plant productivity. Nature.

[CR17] Cafer AM, Rikoon JS (2018). Adoption of new technologies by smallholder farmers: the contributions of extension, research institutes, cooperatives, and access to cash for improving tef production in Ethiopia. Agric Hum values.

[CR18] Corbera E, Roth D, Work C (2019). Climate change policies, natural resources and conflict: implications for development. Clim Policy.

[CR19] Coulibaly JY, Chiputwa B, Nakelse T, Kundhlande G (2017). Adoption of agroforestry and the impact on household food security among farmers in Malawi. Agric Syst.

[CR20] Danquah JA (2015). Analysis of factors influencing farmers’ voluntary participation in reforestation programme in Ghana. For, Trees Livelihoods.

[CR21] Deb S (2020) Traditional Agroforestry Systems of Northeast India. In Socioeconomic and Eco-biological Dimensions in Resource use and Conservation (pp. 103–115). Springer, Cham

[CR22] Dhakal A, Rai RK (2020). Who Adopts Agroforestry in a Subsistence Economy?—Lessons from the Terai of Nepal. Forests.

[CR23] Dhakal A, Cockfield G, Maraseni TN (2015). Deriving an index of adoption rate and assessing factors affecting adoption of an agroforestry-based farming system in Dhanusha District, Nepal. Agrofor Syst.

[CR24] Do H, Luedeling E, Whitney C (2020). Decision analysis of agroforestry options reveals adoption risks for resource-poor farmers. Agron Sustain Dev.

[CR25] Dumont ES, Bonhomme S, Pagella TF, Sinclair FL (2019). Structured stakeholder engagement leads to development of more diverse and inclusive agroforestry options. Exp Agric.

[CR26] Esfandiari M, Khalilabad HRM, Boshrabadi HM, Mehrjerdi MRZ (2020). Factors influencing the use of adaptation strategies to climate change in paddy lands of Kamfiruz, Iran. Land Use Policy.

[CR27] Fisher M, Holden ST, Thierfelder C, Katengeza SP (2018). Awareness and adoption of conservation agriculture in Malawi: what difference can farmer-to-farmer extension make?. Int J Agric Sustain.

[CR28] Fleming A, O’grady AP, Mendham D, England J, Mitchell P, Moroni M, Lyons A (2019). Understanding the values behind farmer perceptions of trees on farms to increase adoption of agroforestry in Australia. Agron Sustain Dev.

[CR29] Gosling E, Knoke T, Reith E, Reyes Cáceres A, Paul C (2021). Which Socioeconomic Conditions Drive the Selection of Agroforestry at the Forest Frontier?. Environ Manag.

[CR30] Hairiah K, Widianto W, Suprayogo D, Van Noordwijk M (2020). Tree roots anchoring and binding soil: reducing landslide risk in Indonesian agroforestry. Land.

[CR31] Hendrickson CY, Corbera E (2015). Participation dynamics and institutional change in the Scolel Té carbon forestry project, Chiapas, Mexico. Geoforum.

[CR32] Hussain J, Zhou K, Akbar M, Raza G, Ali S, Hussain A, Ghulam A (2019). Dependence of rural livelihoods on forest resources in Naltar Valley, a dry temperate mountainous region, Pakistan. Glob Ecol Conserv.

[CR33] Jara-Rojas R, Russy S, Roco L, Fleming-Muñoz D, Engler A (2020). Factors affecting the adoption of agroforestry practices: insights from silvopastoral systems of Colombia. Forests.

[CR34] Jha S, Kaechele H, Sieber S (2021). Factors influencing the adoption of agroforestry by smallholder farmer households in Tanzania: Case studies from Morogoro and Dodoma. Land Use Policy.

[CR35] Kantar MB, Tyl CE, Dorn KM, Zhang X, Jungers JM, Kaser JM,… Wyse DL (2016) Perennial grain and oilseed crops. Annual review of plant biology, 67: 703–729 10.1146/annurev-arplant-043015-112311 26789233

[CR36] Kleinbaum DG, Kupper LL, Nizam A, Rosenberg ES (2013) Applied regression analysis and other multivariable methods. Cengage Learning

[CR37] Koussihouèdé H, Clermont-Dauphin C, Aholoukpè H, Barthès B, Chapuis-Lardy L, Jassogne L, Amadji G (2020). Diversity and socio-economic aspects of oil palm agroforestry systems on the Allada plateau, southern Benin. Agrofor Syst.

[CR38] Läpple D, Van Rensburg T (2011). Adoption of organic farming: Are there differences between early and late adoption?. Ecol Econ.

[CR39] Läpple D, Renwick A, Thorne F (2015). Measuring and understanding the drivers of agricultural innovation: Evidence from Ireland. Food Policy.

[CR40] Lazos‐Chavero E, Zinda J, Bennett‐Curry A, Balvanera P, Bloomfield G, Lindell C, Negra C (2016). Stakeholders and tropical reforestation: challenges, trade‐offs, and strategies in dynamic environments. Biotropica.

[CR41] Le HD, Tran TMA, Thanh Pham H (2021). Key factors influencing forest tree planting decisions of households: A case study in Hoa Binh province, Vietnam. For, Trees Livelihoods.

[CR42] Li Q, Yang W, Li K (2018). Role of social learning in the diffusion of environmentally-friendly agricultural technology in China. Sustain.

[CR43] Li R, Zheng H, Zhang C, Keeler B, Samberg LH, Li C, Ouyang Z (2020). Rural household livelihood and tree plantation dependence in the central mountainous region of Hainan Island, China: implications for poverty alleviation. Forests.

[CR44] Lienhoop N, Brouwer R (2015). Agri-environmental policy valuation: Farmers’ contract design preferences for afforestation schemes. Land Use Policy.

[CR45] Luoranen J, Saksa T, Lappi J (2018). Seedling, planting site and weather factors affecting the success of autumn plantings in Norway spruce and Scots pine seedlings. For Ecol Manag.

[CR46] Mahmood MI, Zubair M (2020). Farmer’s perception of and factors influencing agroforestry practices in the Indus River Basin, Pakistan. Small-scale For.

[CR47] Mishra PK, Rai A, Abdelrahman K, Rai SC, Tiwari A (2022). Land Degradation, Overland Flow, Soil Erosion, and Nutrient Loss in the Eastern Himalayas, India. Land.

[CR48] Nagar B, Rawat S, Pandey R, Kumar M, Alatalo JM (2022) Fuelwood and fodder consumption patterns among agroforestry-practicing smallholder farmers of the lower Himalayas, India. Environ Dev Sustain. 24:5594–5613

[CR49] Nath AJ, Sahoo UK, Giri K, Sileshi GW, Das AK (2020) Incentivizing hill farmers for promoting agroforestry as an alternative to shifting cultivation in Northeast India. In Agroforestry for Degraded Landscapes (pp. 425-444). Springer, Singapore

[CR50] Ndlovu NP, Borrass L (2021). Promises and potentials do not grow trees and crops. A review of institutional and policy research in agroforestry for the Southern African region. Land Use Policy.

[CR51] Nyasimi M, Kimeli P, Sayula G, Radeny M, Kinyangi J, Mungai C (2017). Adoption and dissemination pathways for climate-smart agriculture technologies and practices for climate-resilient livelihoods in Lushoto. Northeast Tanzan Clim.

[CR52] Ochieng J, Afari-Sefa V, Muthoni F, Kansiime M, Hoeschle-Zeledon I, Bekunda M, Thomas D (2021) Adoption of sustainable agricultural technologies for vegetable production in rural Tanzania: trade-offs, complementarities and diffusion. Int. J. Agric. Sustain 20:478–496

[CR53] Ofori E, Griffin T, Yeager E (2020) Duration analyses of precision agriculture technology adoption: what’s influencing farmers’ time-to-adoption decisions? Agri Finance Rev 80:647–664

[CR54] Ota L, Herbohn J, Gregorio N, Harrison S (2020). Reforestation and smallholder livelihoods in the humid tropics. Land Use Policy.

[CR55] Reid R (2017). Developing farmer and community capacity in Agroforestry: is the Australian Master TreeGrower program transferable to other countries?. Agrofor Syst.

[CR56] Rogers EM (2003) Diffusion of innovations, 5th edition. Free Press.

[CR57] Romanova O, Gold MA, Hall DM, Hendrickson MK (2022) Perspectives of Agroforestry Practitioners on Agroforestry Adoption: Case Study of Selected SARE Participants. Rural Sociology. 10.1111/ruso.12463

[CR58] Rosati A, Borek R, Canali S (2021). Agroforestry and organic agriculture. Agrofor Syst.

[CR59] Ruf F, Schroth G (2015) Introduction—Economic and ecological aspects of diversification of tropical tree crops. In Economics and Ecology of Diversification (pp. 1-40). Springer, Dordrecht

[CR60] Simelton E, Dam BV, Catacutan D (2015). Trees and agroforestry for coping with extreme weather events: experiences from northern and central Viet Nam. Agrofor Syst.

[CR61] Singh C, Dorward P, Osbahr H (2016). Developing a holistic approach to the analysis of farmer decision-making: Implications for adaptation policy and practice in developing countries. Land Use Policy.

[CR62] Sukhbaatar G, Ganbaatar B, Jamsran T, Purevragchaa B, Nachin B, Gradel A (2020). Assessment of early survival and growth of planted Scots pine (Pinus sylvestris) seedlings under extreme continental climate conditions of northern Mongolia. J For Res.

[CR63] Tafere SM, Nigussie ZA (2018). The adoption of introduced agroforestry innovations: determinants of a high adoption rate–a case-study from Ethiopia. For, Trees Livelihoods.

[CR64] Trozzo KE, Munsell JF, Chamberlain JL, Gold MA, Niewolny KL (2021). Forest Farming: Who Wants In?. J For.

[CR65] Ullah A, Saqib SE, Kächele H (2022). Determinants of Farmers’ Awareness and Adoption of Extension Recommended Wheat Varieties in the Rainfed Areas of Pakistan. Sustainability.

[CR66] Ullah A, Zeb A, Saqib SE, Kächele H (2022). Landscape co-management and livelihood sustainability: Lessons learned from the billion trees afforestation project in Pakistan. Land Use Policy.

[CR67] Ullah A, Mishra AK, Bavorova M, Kächele H (2022). The effect of COVID-19 pandemic on market integration: Evidence from vegetable farmers in Pakistan.. Int. J. Disaster Risk Reduct.

[CR68] Ullah A, Zeb A, Liu J, Mahmood N, Kächele H (2021). Transhumant pastoralist knowledge of infectious diseases and adoption of alternative land use strategies in the Hindu-Kush Himalayan (HKH) region of Pakistan. Land Use Policy.

[CR69] Ullah A, Sam AS, Sathyan AR, Mahmood N, Zeb A, Kächele H (2021). Role of local communities in forest landscape restoration: Key lessons from the Billion Trees Afforestation Project, Pakistan. Sci Total Environ.

[CR70] Ullah A, Zeb A, Saqib SE, Kächele H (2022a) Constraints to agroforestry diffusion under the Billion Trees Afforestation Project (BTAP), Pakistan: policy recommendations for 10-BTAP. Environ Sci Pollut Res 29:68757–68775 10.1007/s11356-022-20661-9 PMC9508197 35551595

[CR71] Valdivia C, Barbieri C, Gold MA (2012). Between forestry and farming: Policy and environmental implications of the barriers to agroforestry adoption. Can J Agric Econ/Rev canadienne d’agroeconomie.

[CR72] Voss RC, Jansen T, Mané B, Shennan C (2021). Encouraging technology adoption using ICTs and farm trials in Senegal: Lessons for gender equity and scaled impact. World Dev.

[CR73] Wainaina P, Tongruksawattana S, Qaim M (2016). Tradeoffs and complementarities in the adoption of improved seeds, fertilizer, and natural resource management technologies in Kenya. Agric Econ.

[CR74] Wambugu C, Place F, Franzel S (2011). Research, development and scaling-up the adoption of fodder shrub innovations in East Africa. Int J Agric Sustain.

[CR75] Yadav LP, Smith D, Aziz AA, Thuy CTL, Thao HX, Le HH, Vagneron I (2021). Can traders help farmers transition towards more sustainable maize based farming systems? Evidence from the Lao-Vietnamese border. Int J Agric Sustain.

[CR76] Zada M, Zada S, Ali M, Zhang Y, Begum A, Han H, Vega-Muñoz A (2021). Development of local economy through the strengthening of small-medium-sized forest enterprises in KPK, Pakistan. Sustainability.

[CR77] Zada M, Zada S, Ali M, Zhang Y, Begum A, Han H, Araya-Castillo L (2022). Contribution of Small-Scale Agroforestry to Local Economic Development and Livelihood Resilience: Evidence from Khyber Pakhtunkhwa Province (KPK), Pakistan. Land.

[CR78] Zeb A, Armstrong GW, Hamann A (2019). Forest conversion by the indigenous Kalasha of Pakistan: A household level analysis of socioeconomic drivers. Glob Environ Change.

